# A potential role of PI3K inhibition in radiotherapy of glioblastoma multiforme

**DOI:** 10.1186/2194-7791-2-S1-A18

**Published:** 2015-07-01

**Authors:** Julia Langhans, Lukas Schneele, Klaus-Michael Debatin, Mike-Andrew Westhoff

**Affiliations:** Department of Pediatrics and Adolescent Medicine, University Medical Center Ulm, Ulm, Germany

## Meeting abstract

Glioblastoma multiforme (GBM) is the most common tumour of the central nervous system in adults and while it is not frequently considered a paediatric malignancies, it is in terms of 'years of life lost' among the most devastating cancers in children and adolescences. The current standard of care consists of tumour resection followed by radiotherapy and a course of Temozolomide. However, despite intense efforts, the mean patient survival of 12-15 months has not significantly improved over the last two decades.

While the PI3K/Akt/mTOR 'survival' pathway is aberrantly activated in almost 90% of GBMs and despite promising pre-clinical results, pharmacological inhibitors of this signalling cascade have so far not fulfilled their clinical potential.

In this study we use different cell populations cultured directly from primary-derived patient material (so-called cancer stem cells, as well as freshly differentiated cells) to investigate the combination of a pharmacological inhibitor of PI3K, GDC-0941, and clinically relevant doses of radiation. While the induction of apoptosis and the retardation of proliferation is enhanced by the combination treatment in a statistically significant manner, the overall effect is lacklustre. However, importantly, the enhanced motility induced in differentiated GBM cells after radiation can be blocked by PI3K inhibition, while neither radiation, nor GDC-0941 had an effect on the motility of the highly motile cancer stem cells. These data suggest that the role of Pi3K-mediated signalling and the primary mode of movement difference in genetically identical, but epigenetically different GBM cells. We therefore propose that the key role of PI3K inhibitors in combination therapy might lie not in their ability to enhance apoptosis induction, but in preventing tumour invasion in a subpopulation of tumour cells.

